# Comparison of Visual Examination and Magnification with DIAGNOdent for Detection of Smooth Surface Initial Carious Lesion—Dry and Wet Conditions

**DOI:** 10.5005/jp-journals-10005-1588

**Published:** 2019

**Authors:** Noopur Gupta, Meera Sandhu, Vinod Sachdev, Pulkit Jhingan

**Affiliations:** 1–4 Department of Pedodontics and Preventive Dentistry, ITS-CDSR, Ghaziabad, Uttar Pradesh, India

**Keywords:** DIAGNOdent, Magnifying loupes, Reliability, White spot lesion

## Abstract

**Objective:**

The aim of this study was to assess the effectiveness and reliability of magnification, DIAGNOdent in detection of smooth surface white spot lesions.

**Study design:**

Three hundred children aged 5–10 years were examined by two examiners for presence of smooth surface white spot lesions using naked eye and magnifying loupes in wet and dry conditions followed by DIAGNOdent analysis. Data was analysed using Cohen's kappa coefficient, Friedman test and Paired *t* test. Accuracy was assessed by sensitivity and positive predicted values.

**Results:**

Significant difference was found between naked eyes and magnifying loupes with and without air drying. While insignificant difference was found between DIAGNOdent and loupes.

**Conclusion:**

Magnifying loupes with air drying is an effective method in detection of smooth surface white spot lesion.

**Clinical significance:**

With the increased knowledge about the pathogenesis of dental caries and its ability to be remineralisable if detected early, makes it all the more important for the clinician to be vigilant in detection of early lesion to prevent the avoidable restorative approach for the same. Incorporation of magnification to routine dental examination by general dentist can help in early diagnosis and treatment of dental decay. This could prevent further progression of dental caries and reduces the incidence of tooth decay

**How to cite this article:**

Gupta N, Sandhu M, *et al.* Comparison of Visual Examination and Magnification with DIAGNOdent for Detection of Smooth Surface Initial Carious Lesion—Dry and Wet Conditions. Int J Clin Pediatr Dent 2019;12(1):37–41.

## INTRODUCTION

Alternating demineralization and remineralization occurs dynamically in the oral cavity on tooth surface, imbalance of which is the cause of the dental caries. The first visible evidence of dental caries is white spot lesions.^1^ The clinical characteristics of these lesions include:^2^

- Loss of normal translucency of enamel because of altered light properties with a chalky white appearance, particularly when dehydrated;
- A fragile surface layer susceptible to damage from probing, particularly in pits and fissures;
- Increased porosity, particularly of the subsurface, with increased potential for uptake of stain;
- Reduced density of subsurface, which may be detectable radiographically, with transillumination or with modern laser detecting devices;
- A potential for remineralization, with an increased resistance to acid challenge particularly with the use of enhanced remineralization treatments.

Early diagnosis of these white spot lesions is of crucial importance for the survival of teeth, as the occurrence of cavitation as well as further circular spreading of lesions within a few weeks may result in loss of the tooth. Examination of the lesion using the conventional technique may cause damage to the smooth surface lesion and examining of color changes may be confounded by the uptake of colored substances within remineralizing tooth structure.^3^ Early detection of white spot lesions is often missed by a visual examination, as an unaided eye with 20/20 vision can resolve two lines 0.2 mm apart. Magnification allows a resolution of up to 0.05 mm, which if used for detection of white spot lesions, reduces the chances of undetection.^4^ Magnifying loupes used in a dental setup when used in dry conditions provide better specificity in the detection of early carious lesions.^5^

Presently, there is a wide range of detection systems including: quantitative light-induced fluorescence, digital imaging fiber-optic transillumination, and laser fluorescence.^3^ DIAGNOdent (laser fluorescence) is selected as the standard as the other advanced detection aids are overpriced.

Hence, this study was conducted with the aim to evaluate the effectiveness of magnification in the detection of white spot lesions on smooth surfaces of relatively intact teeth with naked eyes or with the use of magnifying loupes, with or without air drying, and further, to correlate the findings with a DIAGNOdent pen.

## MATERIALS AND METHOD

The present cross-sectional study was conducted in the Department of Pedodontics and Preventive Dentistry, ITS Center for Dental Studies and Research, Ghaziabad, Uttar Pradesh, India, and was approved by the ethical committee of the same institution. The study was performed on 300 children aged between 5 years and 10 years who had visually intact caries-free buccal surface of teeth. Children and their parents were informed about the study and a signed consent was taken from their parents. Before recording the readings thorough oral prophylaxis was done.

**Fig. 1:**
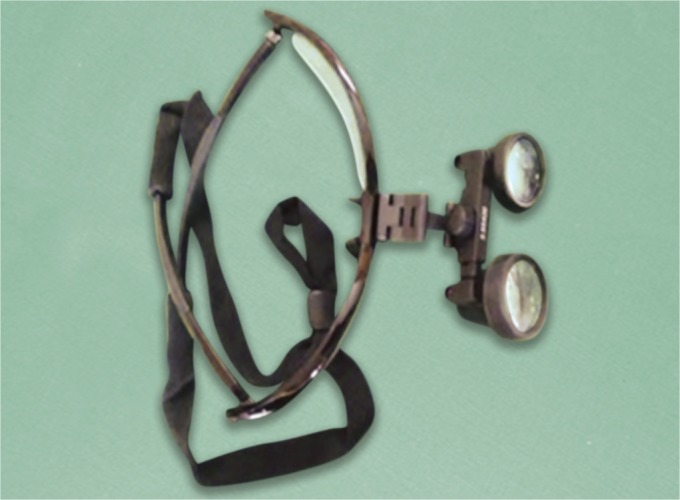
Magnifying loupes

**Fig. 2:**
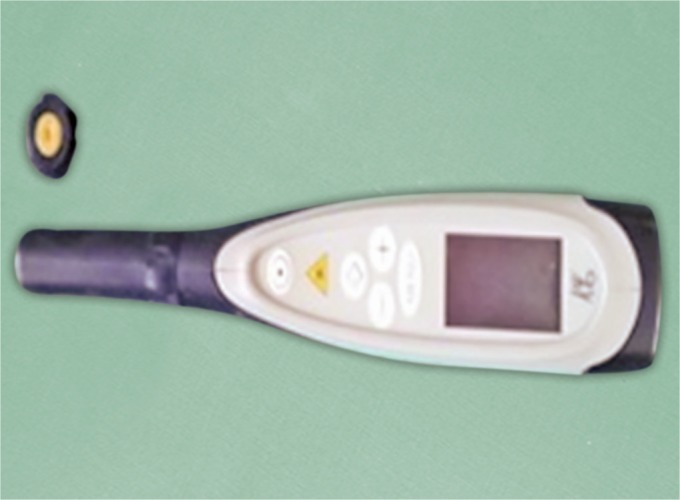
DIAGNOdent

Assessment was done using three diagnostic methods that are naked eye, magnifying loupes (Fig. 1) and DIAGNOdent (Fig. 2) with inter examiner blinding using two examiners calibrated for International Caries Detection and Assessment System (ICDAS) coding.

**Procedure 1:** Buccal surfaces of all samples observed before air drying

1(a) with naked eye
1(b) using loupes (Amtech)

**Procedure 2:** Buccal surfaces of all samples observed after air drying

2(a) with naked eye
2(b) using loupes (Amtech)

**Procedure 3:** Buccal surfaces of all samples observed with DIAGNOdent, which was taken as a standard

### Scoring Criteria

International caries detection and assessment system scoring criteria^6^ with codes 0, 1, and 2 was included in the study [code 0: sound, code 1: first visual change in enamel (seen only after prolonged air drying), code 2: distinct visual change in enamel].

### Statistical Analysis

Data was compiled and statistical analysis was done using SPSS version 20.0. The agreement between the various diagnostic techniques was analyzed by the Friedman test. A paired *t* test was used to compare the scores, before and after air drying within each technique and for two independent groups. Reproducibility was measured by Cohen kappa scores, values of which range from 0 for less than chance agreement to 1 for almost perfect agreement. The level of significance *p* < 0.05.

## RESULTS

### Results of Statistical Analysis

The Friedman test was applied to determine the distribution of mean ranks for all the diagnostic technique results (Table 1). A highly significant difference was found between all the diagnostic methods *p* < 0.05.

The distribution of means ± standard deviation of scores by different techniques before and after air drying are given in Table 2. We found that the mean score (0.99 ± 1) of group 2b (after air drying with magnifying loupes (4.2× magnification) was maximum on applying the paired *t* test. We found that the mean difference of the mean scores between techniques 1(a) and 2(a), 1(b) and 2(b), 1(a) and 1(b), 1(a) and 3, 1(b) and 3, 2(a) and 2(b), and 2(a) and 3 was significant (*p* < 0.05).

Table 3 presents reproducibility and comparison of agreement among the various caries detection techniques using Cohen kappa test. A significant kappa score of 0.706 and 0.294 between 1(a) before air drying with naked eye, –2(a) after air drying with naked eye, and 1(a) before air drying with naked eye and –1(b) before air drying with magnifying loupes (4.2× magnification was seen (*p* = 0.000)) showing significant reproducibility for the given diagnostic techniques as all the teeth included in the study have white spot lesions (true positive). So, there are no false positive or false negative findings. Hence, specificity cannot be calculated.

**Fig. 3:**
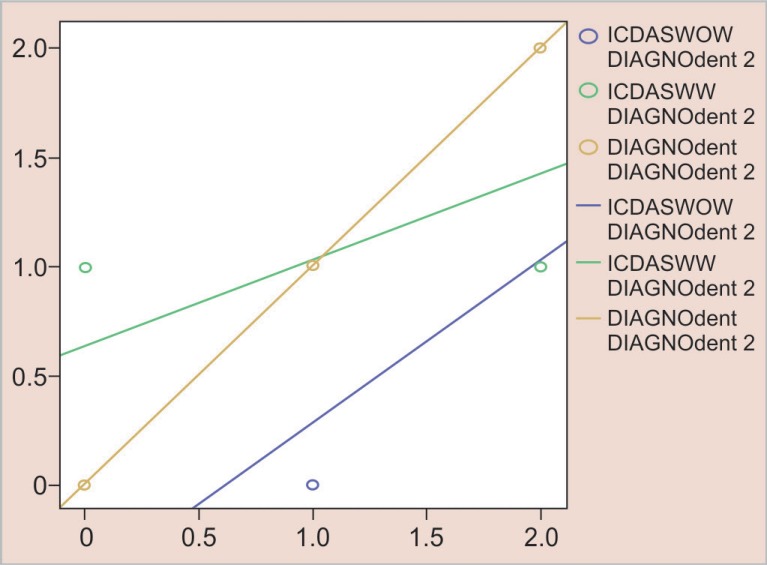
Number of lesions detected by DIAGNOdent, magnifying loupes, and naked eye in dry conditions

Figure 3 represents the number of white spot lesions detected by magnifying loupes and naked eye in dry conditions compared to DIAGNOdent. In dry conditions, detection of white spot lesions by magnifying loupes was more related to that with DIAGNOdent than that with naked eye.

**Fig. 4:**
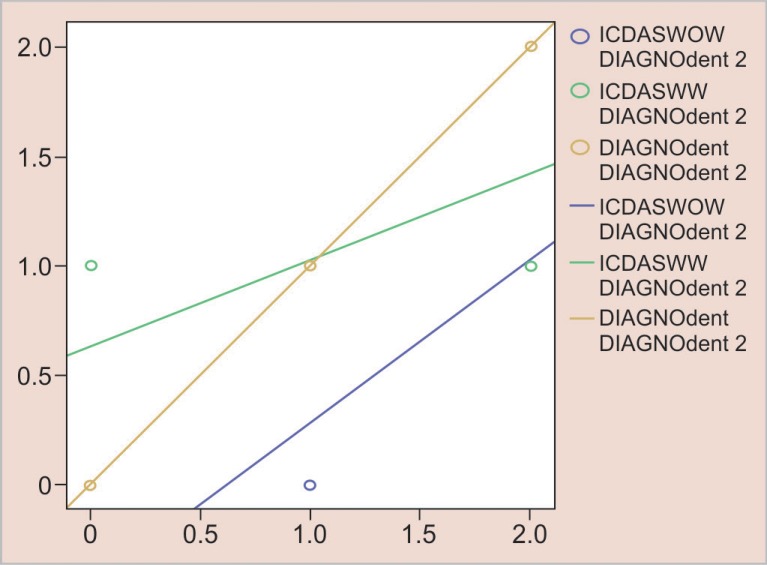
Number of lesions detected by DIAGNOdent, magnifying loupes, and naked eye in wet conditions

Figure 4 represents the number of white spot lesions detected by magnifying loupes and naked eye in wet conditions compared to DIAGOdent. A similar pattern was observed in wet conditions as detection of the lesions in wet conditions by magnifying loupes was more related to DIAGNOdent compared to the naked eye.

On comparison of both the graphs, naked eye detection of lesions was more related to that of DIAGNOdent in dry conditions than in wet conditions.

## DISCUSSION

In the present study, DIAGNOdent was used as the standard for the detection of dental caries. It is one of the newer diagnostic aids, developed by Hibst and Gall in 1998 which is based on the principle of laser fluorescence. It utilizes a 655 nm 1 mW laser diode excitation light source that is modulated to differentiate it from ambient light. The light is transmitted through a descending optical fiber to a hand-held probe. The probe is placed close to the measured surface, thereby illuminating it with the laser light. Carious tooth structures emit fluorescence above 680 nm when encountering this light and this fluorescence is detected and quantified by the drying and distribution (DD) unit as a number between 0 and 99.^7^

**Table 1:** Distribution of mean ranks after applying Friedman test for all the diagnostic techniques

*Diagnostic techniques*	*Mean rank*	*N*	*Chi square*	*Df*	*p value*
1(a). Before air drying with naked eye	2.18				
1(b). Before air drying with magnifying loupes (4.2× magnification	3.13				
2(a). After air drying with naked eye	2.56	100	161.237	4	0.000
2(b). After air drying with magnifying loupes (4.2× magnification	3.56				
3. DIAGNOdent	3.58				

* Significant *p* value <0.05

**Table 2:** Comparison between various diagnostic techniques before and after air drying using *t* test

*Diagnostic method*	*N*	*Mean ± Std deviation*	*p value*
1(a). Before air drying with naked eye	100	0.440 ± 0.499	0.000
2(a). After air drying with naked eye		0.59 ± 0.494	
1(b). Before air drying with magnifying loupes (4.2× magnification)		0.82 ± 0.386	0.000
2(b). After air drying with magnifying loupes (4.2× magnification		0.99 ± 0.100	
1(a). Before air drying with naked eye		0.44 ± 0.499	0.000
1(b). Before air drying with magnifying loupes (4.2× magnification		0.82 ± 0.386	
1(a). Before air drying with naked eye		0.44 ± 0.499	0.000
3. DIAGNOdent		1.00 ± 0.000	
1(b). Before air drying with magnifying loupes (4.2× magnification		0.82 ± 0.386	0.000
3. DIAGNOdent		1.00 ± 0.000	
2(a). After air drying with naked eye		0.59 ± 0.494	0.000
2(b). After air drying with magnifying loupes (4.2× magnification)		0.99 ± 0.100	
2(a). After air drying with naked eye		0.59 ± 0.494	0.000
3. DIAGNOdent		1.00 ± 0.000	
2(b). After air drying with magnifying loupes (4.2× magnification		0.99 ± 0.100	0.314
3. DIAGNOdent		1.00 ± 0.000	

* Significant *p* value <0.05

**Table 3:** Sensitivities and positive predictive values of various diagnostic techniques (compared with DIAGNOdent)

*Diagnostic techniques*	*Sensitivity (%)*	*Positive predictive value (%)*
1(a)	44	100
2(a)	59	100
1(b)	82	100
2(b)	99	100

The sensitivity signifies the ability to detect the presence of the disease. While, specificity denotes the ability to detect the absence of the disease. DIAGNOdent has been proved by various authors as a highly sensitive diagnostic aid for detection of early lesions.^8–10^ Thus, the present study aimed to evaluate the effectiveness of air drying and magnifying loupes in the detection of white spot lesions compared with DIAGNOdent.

In the present study, a significant difference was observed on comparison of naked eye with magnifying loupes in wet and dry condition techniques (*p* < 0.05). Similar results were obtained by Pinelli et al. who observed that air drying the tooth surface before examination increases the reproducibility of caries detection rate using DIAGNOdent and also recommended drying of tooth surface for 10 seconds to assure reliability in diagnosis of dental caries.^11^

The results of the present study also showed that air drying can further improve the specificity of the visual examination as the presence of saliva can mask the early carious lesions due to difference in the refractive index of air and water. Shi et al. reported similar results and found a difference in the detection of white spot lesions in wet and dry conditions on occlusal surfaces.^9^ Braga et al. reported that there are 29 different visual criteria for detection of carious lesion but only half of the technologies recommend drying of tooth surface before examination, which if not included increases the risk of missing lesions which can be detected by the naked eye.^12^

In the present study, results showed that the sensitivity of magnifying loupes was 99% and with naked eye was 82%. Similar results were obtained by Goel et al. in an *in vitro* study reporting 97.2% sensitivity of magnifying loupes when used in dry conditions while that with naked eye was 66.7%.^5^ Pinelli et al. reported similar results and observed that air drying the tooth surface before examination increases the reproducibility of caries detection rate using diagnodent.^11^ Erten et al. evaluated the efficiency of unaided visual examination, operating microscope and intraoral camera in the detection of dental caries and concluded that operating microscope improved caries detection as compared to visual examination alone.^13^

Zahra et al. evaluated the efficacy of DIAGNOdent in the detection of demineralization and remineralization of smooth surface caries in an *in vitro* study and concluded that DIAGNOdent is not useful for detection of remineralization which might be because of use of different remineralization solutions, artificial saliva, and other maintenance solutions. There are various other factors which influence the detection of caries by DIAGNOdent like the presence of plaque, calculus, food deposits, toothpaste, prophylaxis paste, and stains which could give false-positive readings.^14^

The general dental practitioner and pediatric dentist are in a unique position to identify and distinguish between seemingly innocuous conditions that may be normal physiological conditions or early signs of dental decay. Early detection of these lesions ensures a high likelihood of successful therapeutic outcome primarily by reversing the condition.

Various methods available for caries diagnosis are digital substraction radiography, fiber-optic transillumination, DIAGNOdent, electrical caries monitor, quantitative light induced fluorescence, optical coherence tomography, and cone beam-computed tomography.^15,16^ Digital subtraction radiography is technique sensitive and also very senstitive to physical noise occurring in the radiograph causing errors in results. Cortes et al. reported that FOTI is as accurate as the visual method in the detection of caries but authors have also reported about its low sensitivity.^17^ While quantitative light induced fluorescence is a complicated method for use in examination. On the other hand, DIAGNOdent and cone beam computed tomography are expensive. Peker et al. in an *in vitro* study found that for detection of proximal lesions the efficiency of operating microscope was statistically equal to that of unaided visual examination and lower than that of film and digital radiography.^18^

All these methods are either expensive or technique sensitive leaving magnification as a better alternative to provide easier and accurate caries detection. Operating microscope is not economical but instead is technique sensitive. Hence, use of magnifying loupes as daily diagnostic aids provides an economical, accurate and easier method for diagnosing innocuous early carious lesions.

In the present study, only buccal surfaces were examined for the presence of white spot lesions. Further research is required to overcome the potential sources of errors in the clinical conditions.

## CONCLUSION

From the results of the present study the following can be concluded:

- Visual examination after drying has high sensitivity but low specificity. While, magnifying loupes in dry and wet conditions has shown the highest sensitivity but has low specificity as compared to DIAGNOdent.
- Air drying with loupes is a highly effective and reliable method for detection of white spot lesions.

## CLINICAL RELEVANCE

Increased knowledge on the pathogenesis of dental caries and its ability to be remineralizable if detected early makes it all the more important for the clinician to be vigilant in the detection of early lesions to prevent the avoidable restorative approach for the same. Methods for testing these factors and for early detection of dental caries have become sophisticated and should be incorporated into routine dental practices.

Incorporation of magnification into routine dental examination by a general dentist can help in early diagnosis and treatment of dental decay. This could prevent further progression of dental caries and reduce the incidence of tooth decay.

There are various factors which lead to cavitation and we also have different testing methodologies available for them. These testing and detecting systems should hence be routinely used for identification of the susceptible patient long before cavitation becomes evident.^2^
